# Association between dynapenic abdominal obesity and arthritis among the middle-aged and older Chinese: a longitudinal study

**DOI:** 10.1007/s40520-024-02847-y

**Published:** 2024-10-05

**Authors:** Shengliang Zhou, Naijia Luo, Haibo Si, Wacili Da, Yuan Liu, Limin Wu, Mingyang Li, Bin Shen

**Affiliations:** Department of Orthopedic Surgery and Orthopedic Research Institute, West China Hospital, Sichuan University, Chengdu, 610041 China

**Keywords:** Arthritis, Dynapenia, Abdominal obesity, CHARLS

## Abstract

**Background:**

This study aimed to assess the longitudinal association between dynapenic abdominal obesity and new-onset arthritis among the middle-aged and older Chinese population.

**Methods:**

We included 6863 participants from the 2011 and 2015 waves of the China Health and Retirement Longitudinal Study (CHARLS). Dynapenia was defined as handgrip strength < 28 kg for males, and < 18 kg for females. Abdominal obesity was defined as a waist circumference ≥ 90 cm for males and ≥ 85 cm for females. Based on the definitions, all participants were divided into four groups: no dynapenia and no abdominal obesity (ND/NAO), abdominal obesity alone (ND/AO), dynapenia alone (D/NAO), and dynapenia and abdominal obesity (D/AO). The association between dynapenic abdominal obesity and new-onset arthritis was assessed by sex using the Poisson regression models.

**Results:**

After a four-year follow-up, 1272 (18.53%) participants reported new-onset arthritis. Those in the D/AO group had a significantly increased risk of new-onset arthritis compared to those in the ND/NAO group (adjusted relative risk (RR): 1.34, 95% confidence interval (CI): 1.01–1.77). In females, the ND/AO (RR: 1.21, 95% CI: 1.03–1.43) and D/AO (RR: 1.39, 95% CI: 1.01–1.93) groups were associated with a higher risk of arthritis. This significant association was not observed in males.

**Conclusions:**

Our results indicated that the combined effect of dynapenia and abdominal obesity significantly increased the risk of new-onset arthritis in females, but this association was not observed in males.

**Supplementary Information:**

The online version contains supplementary material available at 10.1007/s40520-024-02847-y.

## Introduction

Arthritis, encompassing a range of conditions characterized by joint pain and inflammation, is a leading cause of disability worldwide [1]. Arthritis not only causes considerable pain but also leads to significant activity limitations and a diminished quality of life [2–4]. The incidence of arthritis increases markedly with age, particularly after 45 years. Data from 2010 to 2012 revealed that 22.7% of the US population had doctor-diagnosed arthritis, with projections suggesting a 49% increase in prevalence by 2040 [4]. The economic burden of arthritis is profound; in 2013, it was responsible for over $300 billion in medical expenditures and lost earnings in the US [5]. Similarly, in China, arthritis presented a significant health challenge, with a prevalence of 31.4% reported in 2014 [6]. As the population in many developing countries, particularly in China, continues to age, the burden of arthritis is projected to rise significantly [7]. Thus, identifying and addressing modifiable risk factors for arthritis is essential to mitigate its impact and improve public health outcomes.

Dynapenic abdominal obesity, a complex phenotype characterized by the concurrence of loss of muscle strength (dynapenia) and abdominal obesity, has increasingly been recognized as a significant risk factor affecting various health outcomes of aging populations, such as disability, hospitalization and mortality [8–10]. This phenotype is particularly critical because the loss of muscle strength contributes to fat accumulation, a process potentially driven by such as inflammation, oxidative stress, and insulin resistance [11, 12]. These underlying mechanisms, which contribute to dynapenic abdominal obesity, also play a crucial role in the pathogenesis of arthritis, suggesting a possible link between dynapenic abdominal obesity and the development and progression of arthritis [13, 14]. Prior research indicated that body composition-based obesity and sarcopenic obesity were associated with the risk of knee osteoarthritis [15]. Unlike sarcopenic obesity, which involves the loss of muscle mass alongside increased fat, dynapenic abdominal obesity primarily affects strength rather than muscle mass. The muscle strength has been increasingly recognized as a more effective indicator of sarcopenia than muscle mass [16]. Despite these insights, the effects of dynapenic abdominal obesity on arthritis have not been thoroughly investigated. Therefore, to evaluate the association between dynapenic abdominal obesity and new-onset arthritis, we conducted this longitudinal study among middle-aged and older adults, using data from the China Health and Retirement Longitudinal Study (CHARLS).

## Methods

### Study population

The CHARLS is a comprehensive and ongoing survey that targets a nationally representative cohort of the Chinese population aged 45 and older. Data collection was performed via face-to-face interviews using a computer-assisted personal interviewing (CAPI) technique, conducted by thoroughly trained interviewers. The baseline survey was conducted in 2011, and the subsequent follow-up surveys were performed every two years. Comprehensive details on CHARLS have been extensively documented in prior studies [17]. The CHARLS has been ethically approved by the Ethical Review Committee of Peking University (approval number: IRB00001052-11015), and the written informed consents were secured from all participants in the CHARLS. The performance of CHARLS adhered to the principles of the 1964 Declaration of Helsinki. The current study followed the Strengthening the Reporting of Observational Studies in Epidemiology (STROBE) reporting guidelines.

The data of this study was from CHARLS 2011 and 2015. A total of 17,708 participants were included in the CHARLS 2011. Participants aged < 45 or missing age information (*n* = 391), missing sex information (*n* = 12), missing information on arthritis (*n* = 161), and missing information on dynapenic abdominal obesity status (*n* = 4079). In the baseline survey, 13,065 participants had complete information. Participants with arthritis at baseline (*n* = 4495), and missing the information in the follow-up (*n* = 1707) were further excluded. Finally, a total of 6863 participants were included in the final analysis.

### Assessment of Dynapenia, abdominal obesity, and dynapenic abdominal obesity

Dynapenia, defined as low muscle strength, was assessed using handgrip strength, which was measured with a mechanical spring-type dynamometer (YuejianTM WL-1000, Nantong, China) [17]. Each participant underwent two measurements for each hand, with the maximum value of the dominant handgrip strength used for analysis. Dynapenia was defined as handgrip strength < 28 kg for males, and < 18 kg for females [18]. The waist circumference was measured using a soft measuring tape placed over the clothing around the waist at the level of the navel [17]. The abdominal obesity was defined as a waist circumference ≥ 90 cm for males and ≥ 85 cm for females [19].

Participants were categorized into four distinct groups based on their dynapenia and abdominal obesity status: no dynapenia and no abdominal obesity (ND/NAO), abdominal obesity alone (ND/AO), dynapenia alone (D/NAO), and dynapenia and abdominal obesity (D/AO).

### Assessment of arthritis

The diagnosis of arthritis was based on self-report. All participants were queried: “Have you been diagnosed with arthritis or rheumatism by a doctor?”. Those who responded “yes” were classified as having arthritis.

### Adjustment variables

In this study, a range of baseline variables were selected as adjustment variables in this study, including age, sex, residence (urban and rural), educational level (primary school or below, middle school and high school or above), marital status (married and living with spouse and others), drinking status (current/ever/never drinking), and smoking status (current/ever/never smoking). We also selected some chronic diseases variables based on existing literature [20–23]. Hypertension was defined based on either a self-reported diagnosis by a physician or meeting the clinical criteria of systolic blood pressure (BP) of 140 mmHg or higher, or a diastolic BP of 90 mmHg or higher. Diabetes mellitus (DM) was diagnosed based on a self-report of physician-diagnosed DM, or fasting glucose levels of 7 mmol/L or higher, random glucose levels of 11.1 mmol/L or higher, or glycosylated hemoglobin (HbA1c) levels of 6.5% or higher. Depression was defined as a score of 12 or higher on the 10-item Center for Epidemiologic Studies Depression Scale (CES-D). Cardiovascular disease, pulmonary disease, and kidney disease were identified by the physician-diagnosed history.

### Statistical analysis

Given the sex-specific definitions of dynapenia and abdominal obesity, all analyses were conducted separately for males and females. Continuous variables were presented as means ± standard deviations (SDs), while categorical variables were expressed as numbers (percentages). Differences in characteristics between groups were evaluated using one-way analysis of variance (ANOVA) and chi-square test for continuous and categorical variables, respectively. The association between dynapenic abdominal obesity and new-onset arthritis was examined using Poisson regression models and the relative risks (RR) with corresponding 95% confidence intervals (CIs) were estimated. Three models were used. Model 1 was a crude model. Model 2 adjusted for age, sex, residence, education levels, and marital status. Model 3 additionally adjusted for drinking status, smoking status, hypertension, DM, depression, cardiovascular, pulmonary, and kidney diseases based on Model 2. To assess the robustness of our findings, we conducted several sensitivity analyses. First, we conducted subgroup analyses stratified by age (median age), residence, hypertension, and DM to explore potential mediators. Second, we further adjusted for serum biomarkers including high-density lipoprotein cholesterol (HDL-C), low-density lipoprotein cholesterol (LDL-C), creatinine, and high-sensitivity C-reactive protein (CRP).

All statistical analyses in this study were conducted by STATA/MP (version 17.0). The threshold for statistical significance was set at a two-sided p-value of less than 0.05.

## Results

### Baseline characteristics

A total of 6863 participants were included in the final analyses, with a mean (SD) age was 58.64 (9.32). Of these participants, 3435 (50.05%) were males and 3428 (49.95%) were females. The baseline characteristics of the sample, stratified by sex, were presented in Table 1. Table 2 detailed the baseline characteristics of males according to dynapenia and abdominal status. The proportions of males in the ND/NAO, ND/AO, D/NAO, and D/AO groups were 61.83%, 28.62%, 7.77%, and 1.78%, respectively. Males in the D/AO group tended to be older, more likely to live in urban areas, have lower education levels, have higher levels of CRP, and have higher prevalences of hypertension, DM, depression, and cardiovascular diseases, compared to those in the ND/NAO group. Table 3 presented the baseline characteristics of females according to the dynapenia and abdominal status. The proportions of females in the ND/NAO, ND/AO, D/NAO, and D/AO groups were 44.05%, 45.71%, 5.46%, and 4.78%, respectively. Similar to males, females in the D/AO group were typically older, more likely to live in urban areas, have lower education levels, have higher levels of CRP, and have higher prevalences of hypertension, DM, and cardiovascular diseases.

**Table 1:** Baseline characteristics of included participants according to sex

	Total	Males	Females	*P*-value
N	6863	3435	3428	
Age, years	58.64 ± 9.32	59.30 ± 9.07	57.97 ± 9.52	< 0.001
Residence, (%)				0.637
Urban	1364 (19.94)	675 (19.71)	689 (20.17)	
Rural	5476 (80.06)	2749 (80.29)	2727 (79.83)	
Marital status, (%)				< 0.001
Married and living with spouse	5816 (84.74)	3048 (88.73)	2768 (80.75)	
Others	1047 (15.26)	387 (11.27)	660 (19.25)	
Education level, (%)				< 0.001
Primary school or below	4546 (66.24)	1926 (56.07)	2620 (76.43)	
Middle school	1509 (21.99)	943 (27.45)	566 (16.51)	
High school or above	808 (11.77)	566 (16.48)	242 (7.06)	
Drinking, (%)				< 0.001
Current drinking	2336 (34.04)	1950 (56.79)	386 (11.26)	
Ever drinking	532 (7.75)	412 (12.00)	120 (3.50)	
Never drinking	3994 (58.20)	1072 (31.22)	2922 (85.24)	
Smoking, (%)				< 0.001
Current drinking	2213 (32.34)	2002 (58.57)	211 (6.16)	
Ever drinking	561 (8.20)	505 (14.77)	56 (1.64)	
Never drinking	4069 (59.46)	911 (26.65)	3158 (92.20)	
HDL-C, mg/dL	50.98 ± 15.30	50.61 ± 16.19	51.34 ± 14.37	0.091
LDL-C, mg/dL	116.03 ± 34.97	111.71 ± 34.05	120.21 ± 35.34	< 0.001
Creatine, mg/dL	0.77 ± 0.18	0.87 ± 0.18	0.68 ± 0.13	< 0.001
CRP, mg/L	2.43 ± 6.88	2.54 ± 7.82	2.32 ± 5.84	0.255
Hypertension, (%)	2666 (38.85)	1292 (37.61)	1374 (40.08)	0.036
Diabetes mellitus, (%)	813 (11.86)	384 (11.19)	429 (12.53)	0.087
Depression, (%)	1450 (21.13)	568 (16.54)	882 (25.73)	< 0.001
Cardiovascular disease, (%)	620 (9.07)	267 (7.80)	353 (10.34)	< 0.001
Pulmonary disease, (%)	528 (7.71)	328 (9.56)	200 (5.85)	< 0.001
Kidney disease, (%)	277 (4.05)	171 (5.00)	106 (3.11)	< 0.001
Handgrip strength, kg	32.99 ± 10.41	39.23 ± 9.13	26.73 ± 7.42	< 0.001
Waist circumference, cm	84.38 ± 12.13	84.23 ± 11.97	84.53 ± 12.28	0.3103
Dynapenia and abdominal obesity				< 0.001
ND/NAO	3634 (52.95)	2124 (61.83)	1510 (44.05)	
ND/AO	2550 (37.16)	983 (28.62)	1567 (45.71)	
D/NAO	454 (6.62)	267 (7.77)	187 (5.46)	
D/AO	225 (3.28)	61 (1.78)	164 (4.78)	
New-onset arthritis, (%)	1272 (18.53)	555 (16.16)	717 (20.92)	< 0.001

Continuous variables are presented as the mean ± standard deviation, and categorical variables are expressed as numbers (percentages)

*Abbreviations*: ND/NAO, no dynapenia and no abdominal obesity; ND/AO, abdominal obesity alone; D/NAO, dynapenia alone; D/AO, dynapenia and abdominal obesit

**Table 2:** Baseline characteristics of included males according to the dynapenia and abdominal obesity status

	ND/NAO	ND/AO	D/NAO	D/AO	*P*-value
N	2124 (61.83)	983 (28.62)	267 (7.77)	61 (1.78)	
Age, years	58.71 ± 8.63	57.87 ± 8.71	67.64 ± 9.10	66.49 ± 7.98	< 0.001
Residence, (%)					< 0.001
Urban	350 (16.54)	277 (28.27)	33 (12.36)	15 (24.59)	
Rural	1766 (83.46)	703 (71.73)	234 (87.64)	46 (75.41)	
Marital status, (%)					< 0.001
Married and living with spouse	1881 (88.56)	904 (91.96)	212 (79.40)	51 (83.61)	
Others	243 (11.44)	79 (8.04)	55 (20.60)	10 (16.39)	
Education level, (%)					< 0.001
Primary school or below	1214 (57.16)	454 (46.19)	214 (80.15)	44 (72.13)	
Middle school	581 (27.35)	315 (32.04)	36 (13.48)	11 (18.03)	
High school or above	329 (15.49)	214 (21.77)	17 (6.37)	6 (9.84)	
Drinking, (%)					< 0.001
Current drinking	1232 (58.03)	573 (58.29)	121 (45.32)	26 (42.62)	
Ever drinking	217 (10.22)	132 (13.43)	52 (19.48)	11 (18.03)	
Never drinking	674 (31.75)	278 (28.28)	94 (35.21)	24 (39.34)	
Smoking, (%)					< 0.001
Current drinking	1341 (63.37)	489 (49.95)	153 (58.17)	19 (31.67)	
Ever drinking	256 (12.10)	191 (19.51)	39 (14.83)	19 (31.67)	
Never drinking	519 (24.53)	299 (30.54)	71 (27.00)	22 (36.67)	
HDL-C, mg/dL	53.68 ± 16.25	43.31 ± 13.37	54.73 ± 16.40	42.78 ± 13.90	< 0.001
LDL-C, mg/dL	110.59 ± 33.07	115.39 ± 36.77	107.81 ± 30.23	109.21 ± 34.83	< 0.001
Creatine, mg/dL	0.85 ± 0.16	0.89 ± 0.18	0.87 ± 0.23	0.90 ± 0.23	< 0.001
CRP, mg/L	2.58 ± 9.11	2.44 ± 4.84	2.17 ± 4.89	4.49 ± 7.48	0.314
Hypertension, (%)	627 (29.52)	533 (54.22)	91 (34.08)	41 (67.21)	< 0.001
Diabetes mellitus, (%)	161 (7.59)	179 (18.21)	29 (10.86)	15 (24.59)	< 0.001
Depression, (%)	348 (16.38)	128 (13.02)	76 (28.46)	16 (26.23)	< 0.001
Cardiovascular disease, (%)	115 (5.43)	114 (11.63)	27 (10.11)	11 (18.03)	< 0.001
Pulmonary disease, (%)	213 (10.04)	68 (6.92)	37 (13.86)	10 (16.39)	0.001
Kidney disease, (%)	106 (5.01)	43 (4.39)	17 (6.37)	5 (8.20)	0.373
Handgrip strength, kg	40.03 ± 7.33	43.02 ± 7.89	22.86 ± 4.15	22.09 ± 4.76	< 0.001
Waist circumference, cm	78.96 ± 9.25	96.88 ± 5.87	76.63 ± 11.13	97.31 ± 5.53	< 0.001
New-onset arthritis, (%)	344 (16.20)	137 (13.94)	61 (22.85)	13 (21.31)	0.004

Continuous variables are presented as the mean ± standard deviation, and categorical variables are expressed as numbers (percentages)

*Abbreviations*: ND/NAO, no dynapenia and no abdominal obesity; ND/AO, abdominal obesity alone; D/NAO, dynapenia alone; D/AO, dynapenia and abdominal obesity; HDL-C, high-density lipoprotein cholesterol; LDL-C, low-density lipoprotein cholesterol; CRP, high-sensitivity C-reactive protein

**Table 3:** Baseline characteristics of included females according to the dynapenia and abdominal obesity status

	ND/NAO	ND/AO	D/NAO	D/AO	*P*-value
N	1510 (44.05)	1567 (45.71)	187 (5.46)	164 (4.78)	
Age, years	56.82 ± 9.08	57.37 ± 8.75	64.64 ± 11.29	66.75 ± 10.77	< 0.001
Residence, (%)					< 0.001
Urban	241 (16.05)	368 (23.54)	40 (21.39)	40 (24.39)	
Rural	1261 (83.95)	1195 (76.46)	147 (78.61)	124 (75.61)	
Marital status, (%)					< 0.001
Married and living with spouse	1217 (80.60)	1319 (84.17)	118 (63.10)	114 (69.51)	
Others	293 (19.40)	248 (15.83)	69 (36.90)	50 (30.49)	
Education level, (%)					< 0.001
Primary school or below	1145 (75.83)	1160 (74.03)	165 (88.24)	150 (91.46)	
Middle school	245 (16.23)	297 (18.95)	15 (8.02)	9 (5.49)	
High school or above	120 (7.95)	110 (7.02)	7 (3.74)	5 (3.05)	
Drinking, (%)					0.617
Current drinking	176 (11.66)	175 (11.17)	20 (10.70)	15 (9.15)	
Ever drinking	52 (3.44)	61 (3.89)	3 (1.60)	4 (2.44)	
Never drinking	1282 (84.90)	1331 (84.94)	164 (87.70)	145 (88.41)	
Smoking, (%)					0.052
Current drinking	102 (6.76)	77 (4.91)	19 (10.22)	13 (7.93)	
Ever drinking	23 (1.53)	27 (1.72)	2 (1.08)	4 (2.44)	
Never drinking	1383 (91.71)	1463 (93.36)	165 (88.71)	147 (89.63)	
HDL-C, mg/dL	55.70 ± 14.25	47.36 ± 13.21	54.95 ± 15.32	45.94 ± 12.37	< 0.001
LDL-C, mg/dL	117.92 ± 32.52	122.11 ± 36.56	120.22 ± 38.98	122.87 ± 42.93	0.031
Creatine, mg/dL	0.68 ± 0.12	0.69 ± 0.13	0.68 ± 0.15	0.70 ± 0.17	0.155
CRP, mg/L	1.71 ± 4.62	2.69 ± 5.63	3.72 ± 13.16	2.87 ± 3.50	< 0.001
Hypertension, (%)	428 (28.34)	762 (48.63)	79 (42.25)	105 (64.02)	< 0.001
Diabetes mellitus, (%)	110 (7.28)	260 (16.61)	22 (11.83)	37 (22.56)	< 0.001
Depression, (%)	392 (25.96)	368 (23.48)	72 (38.50)	50 (30.49)	< 0.001
Cardiovascular disease, (%)	119 (7.92)	187 (11.97)	23 (12.37)	24 (14.63)	< 0.001
Pulmonary disease, (%)	88 (5.85)	79 (5.04)	17 (9.29)	16 (9.76)	0.016
Kidney disease, (%)	45 (3.00)	49 (3.14)	5 (2.70)	7 (4.27)	0.825
Handgrip strength, kg	27.73 ± 6.23	28.68 ± 6.18	13.50 ± 3.54	14.04 ± 3.24	0.001
Waist circumference, cm	75.66 ± 9.99	93.19 ± 6.59	74.95 ± 10.29	94.40 ± 7.22	< 0.001
New-onset arthritis, (%)	283 (18.74)	345 (22.02)	44 (23.53)	45 (27.44)	0.015

Continuous variables are presented as the mean ± standard deviation, and categorical variables are expressed as numbers (percentages)

*Abbreviations*: ND/NAO, no dynapenia and no abdominal obesity; ND/AO, abdominal obesity alone; D/NAO, dynapenia alone; D/AO, dynapenia and abdominal obesity; HDL-C, high-density lipoprotein cholesterol; LDL-C, low-density lipoprotein cholesterol; CRP, high-sensitivity C-reactive protein

### Association between dynapenic abdominal obesity and new-onset arthritis

**Fig. 1:**
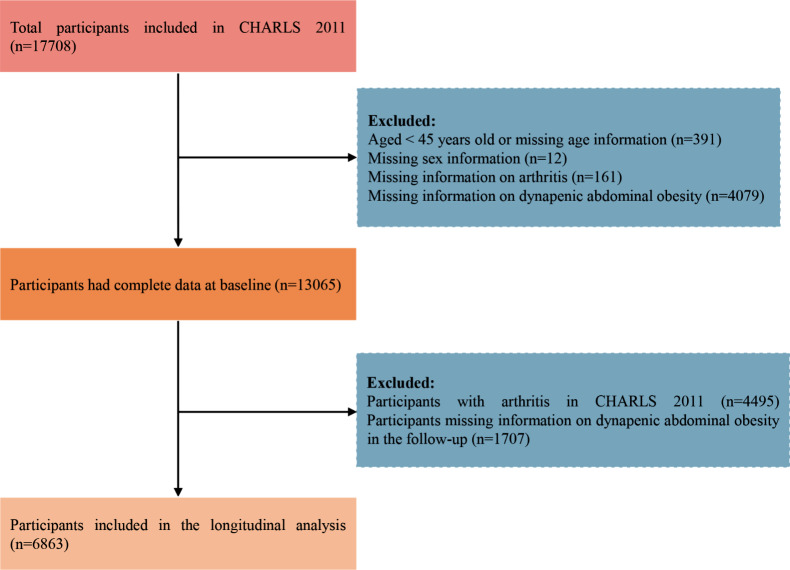
Flowchart of the sample selection process. Abbreviations: CHARLS, China Health and Retirement Longitudinal Study

**Fig. 2:**
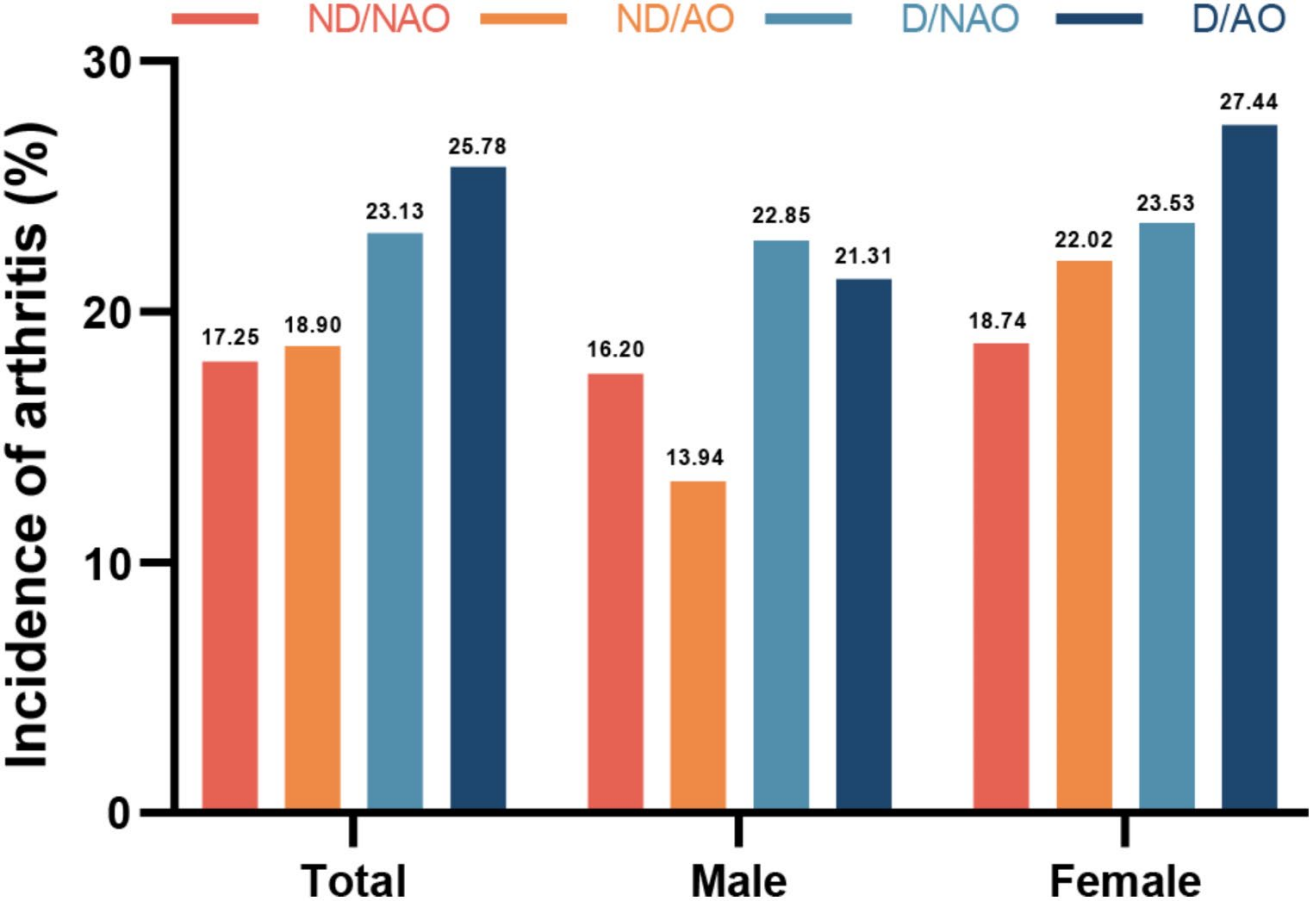
The incidence of arthritis according to the dynapenia and abdominal obesity status. Abbreviations: ND/NAO, no dynapenia and no abdominal obesity; ND/AO, abdominal obesity alone; D/NAO, dynapenia alone; D/AO, dynapenia and abdominal obesity

Over the four-year follow-up, 1272 (18.53%) participants reported new-onset arthritis, of which 555 (16.16%) were males, and 717 (20.92%) were females. The incidence rates of new-onset arthritis stratified by dynapenia and abdominal obesity status, were presented in Figs. 1 and 2. The incidence of arthritis was the highest in the D/AO group, both in the total sample and among females. Compared with participants without new-onset arthritis, those who developed arthritis tended to be older, more likely to be females, live in rural areas, have lower education levels, and have higher prevalences of depression, Cardiovascular and kidney diseases (Table S1). Additionally, these participants typically exhibited weaker handgrip strength (Table S1).

Table 4 showed the association between dynapenic abdominal obesity and new-onset arthritis. Across the total sample, participants in the D/AO group exhibited a significantly higher risk of arthritis compared to those in the ND/NAO group in the fully adjusted model (Model 3, RR:1.34, 95%CI: 1.01–1.77). In males, no significant association between dynapenic abdominal obesity and arthritis was observed. Conversely, in females, both ND/AO (RR:1.21, 95%CI: 1.03–1.43) and D/AO (RR:1.39, 95%CI: 1.01–1.93) groups showed significantly increased risk of arthritis compared to ND/NAO group after adjusting for all covariates.

**Table 4:** Longitudinal association between dynapenic abdominal obesity and new-onset arthritis

	Model 1		Model 2		Model 3		
	RR (95% CI)	*P*-value	RR (95% CI)	*P*-value	RR (95% CI)	*P*-value	
Total sample							
ND/NAO	Ref		Ref		Ref		
ND/AO	1.09 (0.97–1.23)	0.132	1.07 (0.95–1.21)	0.234	1.09 (0.96–1.24)	0.148	
D/NAO	1.34 (1.09–1.64)	0.005	1.27 (1.02–1.57)	0.027	1.20 (0.97–1.49)	0.087	
D/AO	1.49 (1.14–1.95)	0.003	1.34 (1.02–1.77)	0.035	1.34 (1.01–1.77)	0.04	
Males							
ND/NAO	Ref		Ref		Ref		
ND/AO	0.86 (0.70–1.04)	0.137	0.91 (0.74–1.11)	0.367	0.94 (0.76–1.16)	0.574	
D/NAO	1.41 (1.07–1.85)	0.013	1.34 (1.01–1.79)	0.041	1.27 (0.95–1.71)	0.097	
D/AO	1.31 (0.75–2.28)	0.331	1.30 (0.74–2.28)	0.351	1.34 (0.76–2.36)	0.306	
Females							
ND/NAO	Ref		Ref		Ref		
ND/AO	1.17 (1.00-1.37)	0.045	1.20 (1.02–1.41)	0.023	1.21 (1.03–1.43)	0.018	
D/NAO	1.25 (0.91–1.72)	0.16	1.20 (0.87–1.66)	0.26	1.15 (0.83–1.60)	0.393	
D/AO	1.46 (1.06–2.01)	0.018	1.41 (1.02–1.95)	0.036	1.39 (1.01–1.93)	0.047	

Model 1: crude model

Model 2: adjusted for age, sex, residence, marital status, and education levels

Model 3: further adjusted for drinking status, smoking status, hypertension, diabetes mellitus, depression, cardiovascular disease, pulmonary disease, and kidney disease based on Model 2

*Abbreviations*: RR, relative risk; CI, confidence interval; ND/NAO, no dynapenia and no abdominal obesity; ND/AO, abdominal obesity alone; D/NAO, dynapenia alone; D/AO, dynapenia and abdominal obesity

### Sensitive analyses

To further explore potential mediators of the association, subgroup analyses stratified by median age (58 years old), residence, hypertension, DM, and depression were conducted. These analyses were detailed in Table S2. In subgroup analyses, females who were living in rural areas, free from depression were more responsive to the adverse effects of dynapenic abdominal obesity on new-onset arthritis (Table S2). No significant differences were observed in the subgroup analyses among males. After further adjusting for HDL-C, LDL-C, creatinine, and CRP levels, the reanalyzed data showed that the association between dynapenic abdominal obesity and new-onset arthritis did not change significantly (Table S3).

## Discussion

Our longitudinal study using nationally representative data from the CHARLS, investigated the association between dynapenic abdominal obesity and new-onset arthritis. We found that dynapenic abdominal obesity significantly increased the risk of arthritis across the total sample, with a pronounced association observed particularly in females. No significant association was identified in males.

Our findings revealed significant adverse effects of the combination of dynapenia and abdominal obesity on new-onset arthritis, with a pronounced impact observed specifically in females, but not in males. The adverse effects of dynapenia and abdominal obesity on arthritis are generally consistent with existing literature. Previous studies have demonstrated that a higher waist circumference is correlated with an increased risk of arthritis [24, 25]. In addition, recent research in a Korean population found that enhanced handgrip strength was linked with a lower prevalence of arthritis, underscoring the role of muscle strength in joint health [26]. While no study has directly examined the combined effects of dynapenia and abdominal obesity on arthritis, research by Misra et al. on sarcopenic obesity provides indirect support for our findings [15]. This longitudinal study indicated that sarcopenic obesity is associated with an increased risk of knee osteoarthritis, suggesting that similar mechanisms may underlie the impact of dynapenic abdominal obesity on arthritis [15]. Our findings provided new evidence on the effects of dynapenic abdominal obesity on new-onset arthritis among the middle-aged and older Chinese population. Regarding the observed sex differences, Linauskas et al. conducted a prospective study and identified that the positive association between waist circumference and risk of arthritis only existed in females, not in males [24]. In addition, Misra et al. also observed that sarcopenic obesity was associated with an increased risk of knee osteoarthritis in females, rather than in males [15]. These observations underscore the need to consider sex-specific factors in the evaluation of arthritis risk associated with body composition.

The underlying potential mechanisms between dynapenic abdominal obesity and arthritis are still unclear, which may be explained by multiple biological pathways. First, this association could be attributed to chronic sterile low-grade inflammation. The increase of fat mass, especially visceral fat, is known to elevate levels of pro-inflammatory cytokines, such as interleukin-6 (IL-6), tumor necrosis factor-α, and CRP [27]. These cytokines play critical roles in the development and progression of arthritis [28, 29]. Second, dynapenic abdominal obesity has been associated with metabolic disturbances, particularly insulin resistance, which is known to be more prevalent in individuals with arthritis [30]. Moreover, the mechanical stress from the increased body weight and reduced muscle strength may also be a contributing factor to the wear and tear of joints. Further studies are needed to elucidate the precise mechanisms of the association between dynapenic abdominal obesity and arthritis.

In our study, the observed sex differences in the association between dynapenic abdominal obesity and arthritis may be related to hormonal influence. Females generally have higher levels of estrogen, which has notable anti-inflammatory effects [31, 32]. However, after menopause, females experience a rapid decline in estrogen levels, which can lead to an increase in systemic inflammation [33]. This may increase the risk of arthritis. In addition, postmenopausal women are prone to gain more visceral fat, which is associated with higher levels of inflammation and metabolic disturbances that can exacerbate joint degradation [34].

In this study, we demonstrated a novel association between dynapenic abdominal obesity and new-onset arthritis among the middle-aged and older Chinese population, which contributed to the new understanding of how body composition influences joint health in the aging population. Another strength of this study was the use of data from a large and nationally representative sample, which enhanced the generalizability of our findings. However, several limitations should be noted. First, the diagnosis of arthritis in this study was based on self-reported physician diagnoses, which may introduce some degree of bias. In addition, the CHARLS did not include specific classifications for different types of arthritis, such as osteoarthritis or rheumatoid arthritis. Consequently, the results may not directly translate to distinct arthritis types, necessitating caution in their application to specific clinical conditions. Finally, our investigation was confined to the effects of dynapenic abdominal obesity on arthritis within the Chinese population, limiting the generalizability of the findings to other populations.

## Conclusions

In conclusion, our findings revealed a significant association between dynapenic abdominal obesity and an increased risk of new-onset arthritis in females, while no such association was observed in males. These results highlight the importance of addressing not only general obesity but also specific conditions such as dynapenic abdominal obesity in preventive strategies for arthritis.

## Electronic supplementary material

Below is the link to the electronic supplementary material.

Supplementary Material 1

## Data Availability

No datasets were generated or analysed during the current study.

## References

[1] Furner SE, Hootman JM, Helmick CG, Bolen J, Zack MM (2011) Health-related quality of life of US adults with arthritis: analysis of data from the behavioral risk factor surveillance system, 2003, 2005, and 2007. Arthritis Care Res 63(6):788–799 10.1002/acr.20430 21538946

[2] Theis KA, Murphy L, Hootman JM, Helmick CG, Yelin E (2007) Prevalence and correlates of arthritis-attributable work limitation in the US population among persons ages 18–64: 2002 National Health interview Survey Data. Arthritis Rheum 57(3):355–363 17394215 10.1002/art.22622 PMC2875147

[3] Theis KA, Murphy L, Hootman JM, Wilkie R (2013) Social participation restriction among US adults with arthritis: a population-based study using the international classification of functioning, disability and health. Arthritis Care Res 65(7):1059–1069 10.1002/acr.21977 PMC4466902 23401463

[4] Hootman JM, Helmick CG, Barbour KE, Theis KA, Boring MA (2016) Updated projected prevalence of self-reported doctor-diagnosed arthritis and arthritis-attributable activity limitation among US adults, 2015–2040. 68(7):1582–1587 Arthritis & rheumatology (Hoboken, NJ) 10.1002/art.39692 PMC6059375 27015600

[5] Murphy LB, Cisternas MG, Pasta DJ, Helmick CG, Yelin EH (2018) Medical expenditures and earnings losses among US adults with arthritis in 2013. Arthritis Care Res 70(6):869–876 10.1002/acr.23425 28950426

[6] Li C, Liu T, Sun W, Wu L, Zou ZY (2015) Prevalence and risk factors of arthritis in a middle-aged and older Chinese population: the China health and retirement longitudinal study. Rheumatology (Oxford) 54(4):697–706 25288780 10.1093/rheumatology/keu391

[7] Edwards RD (2012) Population aging, the dependency burden, and challenges facing preventive medicine. Prev Med 55(6):533–534 22890023 10.1016/j.ypmed.2012.07.025

[8] da Silva Alexandre T, Scholes S, Ferreira Santos JL, de Oliveira Duarte YA, de Oliveira C (2018) Dynapenic abdominal obesity increases mortality risk among English and Brazilian older adults: a 10-Year Follow-Up of the ELSA and SABE studies. J Nutr Health Aging 22(1):138–14429300433 10.1007/s12603-017-0966-4

[9] Rossi AP, Bianchi L, Volpato S, Bandinelli S, Guralnik J, Zamboni M et al (2017) Dynapenic abdominal obesity as a predictor of worsening disability, hospitalization, and mortality in older adults: results from the InCHIANTI Study. The journals of gerontology Series A, Biological sciences and medical sciences. 72(8):1098–1104 10.1093/gerona/glw203 PMC5861871 28329134

[10] Qian S, Wen Q, Huang T, Chen J, Feng X (2024) Dynapenic abdominal obesity and incident functional disability: results from a nationwide longitudinal study of middle-aged and older adults in China. Arch Gerontol Geriatr 123:105434 38583265 10.1016/j.archger.2024.105434

[11] Axelrod CL, Dantas WS, Kirwan JP (2023) Sarcopenic obesity: emerging mechanisms and therapeutic potential. Metab Clin Exp 146:155639 37380015 10.1016/j.metabol.2023.155639 PMC11448314

[12] Hong SH, Choi KM (2020) Sarcopenic obesity, insulin resistance, and their implications in cardiovascular and metabolic consequences. Int J Mol Sci, 21(2) 10.3390/ijms21020494 PMC7013734 31941015

[13] Riegger J, Schoppa A, Ruths L, Haffner-Luntzer M, Ignatius A (2023) Oxidative stress as a key modulator of cell fate decision in osteoarthritis and osteoporosis: a narrative review. Cell Mol Biol Lett 28(1):76 37777764 10.1186/s11658-023-00489-y PMC10541721

[14] Tripolino C, Ciaffi J, Pucino V, Ruscitti P, van Leeuwen N, Borghi C et al (2021) Insulin signaling in arthritis. Front Immunol 12:672519 33995414 10.3389/fimmu.2021.672519 PMC8119635

[15] Misra D, Fielding RA, Felson DT, Niu J, Brown C, Nevitt M Risk of knee osteoarthritis With obesity, sarcopenic obesity, and sarcopenia. Arthritisrheumatology (, Hoboken et al (2019) NJ), 71(2):232–237 10.1002/art.40692 PMC6374038 30106249

[16] Menant JC, Weber F, Lo J, Sturnieks DL, Close JC, Sachdev PS et al (2017) Strength measures are better than muscle mass measures in predicting health-related outcomes in older people: time to abandon the term Sarcopenia? Osteoporosis international: a journal established as result of cooperation between the European Foundation for Osteoporosis and the National Osteoporosis Foundation of the USA. 28(1):59–70 10.1007/s00198-016-3691-7 27394415

[17] Zhao Y, Hu Y, Smith JP, Strauss J, Yang G (2014) Cohort profile: the China Health and Retirement Longitudinal Study (CHARLS). Int J Epidemiol 43(1):61–68 23243115 10.1093/ije/dys203 PMC3937970

[18] Chen LK, Woo J, Assantachai P, Auyeung TW, Chou MY, Iijima K et al (2020) Asian working group for sarcopenia: 2019 consensus update on sarcopenia diagnosis and treatment. J Am Med Dir Assoc 21(3):300–307e302 32033882 10.1016/j.jamda.2019.12.012

[19] Zhang L, Wang Z, Wang X, Chen Z, Shao L, Tian Y et al (2019) Prevalence of abdominal obesity in China: results from a cross-sectional study of nearly half a million participants. Obes (Silver Spring Md) 27(11):1898–1905 10.1002/oby.22620 31549787

[20] Rehling T, Bjørkman AD, Andersen MB, Ekholm O, Molsted S (2019) Diabetes is associated with musculoskeletal pain, osteoarthritis, osteoporosis, and rheumatoid arthritis. Journal of diabetes research, 2019:6324348 10.1155/2019/6324348 PMC6925775 31886282

[21] Nerurkar L, Siebert S, McInnes IB, Cavanagh J (2019) Rheumatoid arthritis and depression: an inflammatory perspective. Lancet Psychiatry 6(2):164–173 30366684 10.1016/S2215-0366(18)30255-4

[22] Liang X, Chou OHI, Cheung CL, Cheung BMY (2022) Is hypertension associated with arthritis? The United States national health and nutrition examination survey 1999–2018. Ann Med 54(1):1767–1775 35786117 10.1080/07853890.2022.2089911 PMC9258429

[23] England BR, Thiele GM, Anderson DR, Mikuls TR (2018) Increased cardiovascular risk in rheumatoid arthritis: mechanisms and implications. BMJ (Clinical Res ed 361:k1036 10.1136/bmj.k1036 PMC6889899 29685876

[24] Linauskas A, Overvad K, Symmons D, Johansen MB, Stengaard-Pedersen K, de Thurah A (2019) Body fat percentage, waist circumference, and obesity as risk factors for rheumatoid arthritis: a Danish cohort study. Arthritis Care Res 71(6):777–786 10.1002/acr.23694 29975015

[25] Marchand NE, Sparks JA, Tedeschi SK, Malspeis S, Costenbader KH, Karlson EW et al (2021) Abdominal obesity in comparison with general obesity and risk of developing rheumatoid arthritis in women. J Rhuematol 48(2):165–173 10.3899/jrheum.200056 PMC8006183 32669445

[26] Lee J, Lee MG (2019) Associations of handgrip strength with prevalence of rheumatoid arthritis and diabetes mellitus in older adults. J Obes Metabolic Syndrome 28(4):271–277 10.7570/jomes.2019.28.4.271 PMC6939704 31909370

[27] Zamboni M, Mazzali G, Fantin F, Rossi A, Di Francesco V (2008) Sarcopenic obesity: a new category of obesity in the elderly. Nutrition, metabolism, and cardiovascular diseases. NMCD 18(5):388–395 18395429 10.1016/j.numecd.2007.10.002

[28] Pandolfi F, Franza L, Carusi V, Altamura S, Andriollo G, Nucera E (2020) Interleukin-6 in Rheumatoid Arthritis. International journal of molecular sciences, 21(15) 10.3390/ijms21155238 PMC7432115 32718086

[29] Wang T, He C (2018) Pro-inflammatory cytokines: the link between obesity and osteoarthritis. Cytokine Growth Factor Rev 44:38–50 30340925 10.1016/j.cytogfr.2018.10.002

[30] Moellering DR, Smith-Johnston K, Kelley C, Sammy MJ, Benedict J, Brock G et al (2023) Association between skeletal muscle mitochondrial dysfunction and insulin resistance in patients with rheumatoid arthritis: a case-control study. Arthritis Res Therapy 25(1):85 10.1186/s13075-023-03065-z PMC10199606 37210569

[31] Tiidus PM (2001) Oestrogen and sex influence on muscle damage and inflammation: evidence from animal models. Curr Opin Clin Nutr Metab Care 4(6):509–513 11706285 10.1097/00075197-200111000-00008

[32] Viña J, Sastre J, Pallardó FV, Gambini J, Borrás C (2006) Role of mitochondrial oxidative stress to explain the different longevity between genders: protective effect of estrogens. Free Radic Res 40(12):1359–1365 17090425 10.1080/10715760600952851

[33] Cioffi M, Esposito K, Vietri MT, Gazzerro P, D’Auria A, Ardovino I et al (2002) Cytokine pattern in postmenopause. Maturitas 41(3):187–192 11886764 10.1016/s0378-5122(01)00286-9

[34] Kolb H (2022) Obese visceral fat tissue inflammation: from protective to detrimental? BMC Med 20(1):494 36575472 10.1186/s12916-022-02672-y PMC9795790

