# Supplementary material for: Association between dynapenic abdominal obesity and arthritis among the middle-aged and older Chinese: a longitudinal study
Source: Aging Clin Exp Res. 2024 Oct 5;36(1):198. doi: 10.1007/s40520-024-02847-y (PMC11455664; doi:10.1007/s40520-024-02847-y)
Supplement: Supplementary file 1 — Supplementary Material 1 [file 40520_2024_2847_MOESM1_ESM.docx]

**Table S1** Baseline characteristics of participants with and without new-onset arthritis.

|  | **Total** | **New-onset arthritis** | | **P-value** |
| --- | --- | --- | --- | --- |
|  |  | **Yes** | **No** |  |
| N | 6863 | 1272 (18.53) | 5591 (81.47) |  |
| Age, years | 58.64 ± 9.32 | 59.15 ± 9.05 | 58.52 ± 9.38 | 0.027 |
| Sex, (%) |  |  |  | <0.001 |
| Male | 3435 (50.05) | 555 (43.63) | 2880 (51.51) |  |
| Female | 3428 (49.95) | 717 (56.37) | 2711 (48.49) |  |
| Residence, (%) |  |  |  | <0.001 |
| Urban | 1364 (19.94) | 206 (16.23) | 1158 (20.79) |  |
| Rural | 5476 (80.06) | 1063 (83.77) | 4413 (79.23) |  |
| Marital status, (%) |  |  |  | 0.085 |
| Married and living with spouse | 5816 (84.74) | 1058 (83.18) | 4758 (85.10) |  |
| Others | 1047 (15.26) | 214 (16.82) | 833 (14.90) |  |
| Education level, (%) |  |  |  | <0.001 |
| Primary school or below | 4546 (66.24) | 954 (75.00) | 3592 (64.25) |  |
| Middle school | 1509 (21.99) | 214 (16.82) | 1295 (23.16) |  |
| High school or above | 808 (11.77) | 104 (8.18) | 704 (12.59) |  |
| Drinking, (%) |  |  |  | 0.019 |
| Current drinking | 2336 (34.04) | 396 (31.13) | 1940 (34.70) |  |
| Ever drinking | 532 (7.75) | 91 (7.89) | 441 (7.89) |  |
| Never drinking | 3994 (58.20) | 785 (61.71) | 3209 (57.41) |  |
| Smoking, (%) |  |  |  | 0.001 |
| Current drinking | 2213 (32.34) | 378 (29.81) | 1835 (32.91) |  |
| Ever drinking | 561 (8.20) | 82 (6.47) | 479 (8.59) |  |
| Never drinking | 4069 (59.46) | 808 (63.72) | 3261 (58.49) |  |
| HDL-C, mg/dL | 50.98 ± 15.30 | 52.09 ± 15.46 | 50.71 ± 15.25 | 0.01 |
| LDL-C, mg/dL | 116.03 ± 34.97 | 116.00 ± 34.84 | 116.04 ± 35.00 | 0.976 |
| Creatine, mg/dL | 0.77 ± 0.18 | 0.78 ± 0.18 | 0.76 ± 0.17 | 0.04 |
| CRP, mg/L | 2.43 ± 6.88 | 2.48 ± 7.19 | 2.25 ± 5.45 | 0.354 |
| Hypertension, (%) | 2666 (38.85) | 495 (38.92) | 2171 (38.83) | 0.955 |
| Diabetes mellitus, (%) | 813 (11.86) | 153 (12.03) | 660 (11.82) | 0.834 |
| Depression, (%) | 1450 (21.13) | 384 (30.19) | 1066 (19.07) | <0.001 |
| Cardiovascular disease, (%) | 620 (9.07) | 143 (11.29) | 477 (8.56) | 0.002 |
| Pulmonary disease, (%) | 528 (7.71) | 104 (8.21) | 424 (7.60) | 0.462 |
| Kidney disease, (%) | 277 (4.05) | 64 (5.04) | 213 (3.83) | 0.047 |
| Handgrip strength, kg | 32.99 ± 10.41 | 30.95 ± 10.11 | 33.45 ± 10.41 | <0.001 |
| Waist circumference, cm | 84.38 ± 12.13 | 84.86 ± 11.23 | 84.27 ± 12.32 | 0.121 |
| Dynapenia and abdominal obesity |  |  |  | <0.001 |
| ND/NAO | 3634 (52.95) | 627 (49.29) | 3007 (53.78) |  |
| ND/AO | 2550 (37.16) | 482 (37.89) | 2068 (36.99) |  |
| D/NAO | 454 (6.62) | 105 (8.25) | 349 (6.24) |  |
| D/AO | 225 (3.28) | 58 (4.56) | 167 (2.99) |  |

Continuous variables are presented as the mean ± standard deviation, and categorical variables are expressed as numbers (percentages).

Abbreviations: ND/NAO, no dynapenia and no abdominal obesity; ND/AO, abdominal obesity alone; D/NAO, dynapenia alone; D/AO, dynapenia and abdominal obesity

**Table S2** Subgroup analyses of the association between dynapenic abdominal obesity and new-onset arthritis.

|  | **Age** | | **Residence** | | **Hypertension** | | **DM** | | **Depression** | |
| --- | --- | --- | --- | --- | --- | --- | --- | --- | --- | --- |
|  | **<58** | **≥ 58** | **Urban** | **Rural** | **Yes** | **No** | **Yes** | **No** | **Yes** | **No** |
| **Males** |  |  |  |  |  |  |  |  |  |  |
| ND/NAO | Ref | Ref | Ref | Ref | Ref | Ref | Ref | Ref | Ref | Ref |
| ND/AO | 1.02 (0.75-1.39) | 0.87 (0.65-1.17) | 0.94 (0.58-1.52) | 0.93 (0.74-1.18) | 1.02 (0.74-1.40) | 0.85 (0.64-1.14) | 1.01 (0.56-1.81) | 0.92 (0.73-1.15) | 0.92 (0.57-1.46) | 0.94 (0.74-1.20) |
| D/NAO | 0.99 (0.43-2.27) | 1.36 (0.99-1.87) | 1.30 (0.56-3.03) | 1.28 (0.94-1.75) | 1.26 (0.74-2.14) | 1.28 (0.90-1.82) | 0.82 (0.29-2.29) | 1.33 (0.98-1.81) | 1.16 (0.68-1.97) | 1.30 (0.91-1.85) |
| D/AO | 1.18 (0.28-4.86) | 1.41 (0.75-2.63) | 0.87 (0.20-3.79) | 1.52 (0.82-2.80) | 1.39 (0.66-2.89) | 1.40 (0.56-3.45) | 1.05 (0.23-4.71) | 1.45 (0.78-2.68) | 1.36 (0.54-3.42) | 1.29 (0.63-2.66) |
| **Females** |  |  |  |  |  |  |  |  |  |  |
| ND/NAO | Ref | Ref | Ref | Ref | Ref | Ref | Ref | Ref | Ref | Ref |
| ND/AO | 1.33 (1.06-1.67) | 1.09 (0.86-1.38) | 1.24 (0.81-1.91) | 1.21 (1.01-1.44) | 1.37 (1.03-1.82) | 1.15 (0.93-1.41) | 1.61 (0.91-2.85) | 1.19 (1.00-1.41) | 1.18 (0.88-1.57) | 1.21 (0.99-1.48) |
| D/NAO | 1.67 (0.96-2.91) | 0.97 (0.64-1.46) | 0.81 (0.31-2.14) | 1.22 (0.86-1.73) | 1.25 (0.73-2.12) | 1.13 (0.74-1.72) | 1.03 (0.35-3.01) | 1.16 (0.82-1.64) | 1.29 (0.81-2.06) | 1.04 (0.65-1.68) |
| D/AO | 1.50 (0.75-2.97) | 1.27 (0.87-1.87) | 1.08 (0.46-2.53) | 1.48 (1.04-2.12) | 1.45 (0.92-2.27) | 1.41 (0.85-2.34) | 1.45 (0.64-3.28) | 1.40 (0.97-2.02) | 1.07 (0.61-1.91) | 1.58 (1.05-2.36) |

Data are presented as RRs (95%CIs).

The subgroup analyses adjusted for age, sex, residence, marital status, educational level, drinking status, smoking status, hypertension, DM, depression, cardiovascular disease, pulmonary disease, and kidney disease.

Abbreviations: RR, relative risk; CI, confidence interval; ND/NAO, no dynapenia and no abdominal obesity; ND/AO, abdominal obesity alone; D/NAO, dynapenia alone; D/AO, dynapenia and abdominal obesity; DM, diabetes mellitus.

**Table S3** Longitudinal association between dynapenic abdominal obesity and new-onset arthritis after further adjusting for BMI (n=6691) and serum biomarkers (n=4936).

|  | **Model 4** | |
| --- | --- | --- |
|  | **RR (95% CI)** | **P-value** |
| **Total sample** |  |  |
| ND/NAO | Ref |  |
| ND/AO | 1.13 (0.98-1.32) | 0.09 |
| D/NAO | 1.24 (0.97-1.58) | 0.08 |
| D/AO | 1.50 (1.09-2.06) | 0.012 |
| **Males** |  |  |
| ND/NAO | Ref |  |
| ND/AO | 0.95 (0.74-1.22) | 0.720 |
| D/NAO | 1.22 (0.87-1.71) | 0.244 |
| D/AO | 1.40 (0.75-2.61) | 0.289 |
| **Females** |  |  |
| ND/NAO | Ref |  |
| ND/AO | 1.26 (1.04-1.53) | 0.016 |
| D/NAO | 1.28 (0.89-1.84) | 0.176 |
| D/AO | 1.59 (1.09-2.33) | 0.015 |

Data are presented as RRs (95%CIs).

Model 4 adjusted for age, sex, residence, marital status, educational level, drinking status, smoking status, hypertension, DM, depression, cardiovascular disease, pulmonary disease, kidney disease, HDL-C, LDL-C, creatinine, and CRP levels. Abbreviations: RR, relative risk; CI, confidence interval; ND/NAO, no dynapenia and no abdominal obesity; ND/AO, abdominal obesity alone; D/NAO, dynapenia alone; D/AO, dynapenia and abdominal obesity; DM, diabetes mellitus; HDL-C, high-density lipoprotein cholesterol; LDL-C, low-density lipoprotein cholesterol; CRP, high-sensitivity C-reactive protein.

**Table S4** The independent effects of abdominal obesity and dynapenia on new-onset arthritis.

|  | **Model 3** | |
| --- | --- | --- |
|  | **RR (95% CI)** | **P-value** |
| **Abdominal obesity** |  |  |
| Total sample |  |  |
| Yes | 1.09 (0.96-1.23) | 0.147 |
| No | Ref |  |
| Males |  |  |
| Yes | 0.94 (0.76-1.14) | 0.549 |
| No | Ref |  |
| Females |  |  |
| Yes | 1.21 (1.03-1.41) | 0.014 |
| No | Ref |  |

Data are presented as RRs (95%CIs).

Model 3: adjusted for age, sex, residence, marital status, educational level, drinking status, smoking status, hypertension, DM, depression, cardiovascular disease, pulmonary disease, and kidney disease. Abbreviations: RR, relative risk; CI, confidence interval; DM, diabetes mellitus.
